# ANGPTL2 promotes VEGF-A synthesis in human lung cancer and facilitates lymphangiogenesis

**DOI:** 10.18632/aging.204581

**Published:** 2023-03-13

**Authors:** Po-I Liu, Ya-Jing Jiang, An-Chen Chang, Chang-Lun Huang, Yi-Chin Fong, Jeng-Hung Guo, Chun-Lin Liu, Shih-Wei Wang, Ju-Fang Liu, Sunny Li-Yu Chang, Chih-Hsin Tang

**Affiliations:** 1 Department of General Thoracic Surgery, Asia University Hospital, Taichung, Taiwan; 2 Department of Physical Therapy, Asia University, Taichung, Taiwan; 3 Graduate Institute of Biomedical Science, China Medical University, Taichung, Taiwan; 4 Translational Medicine Center, Shin-Kong Wu Ho-Su Memorial Hospital, Taipei, Taiwan; 5 Division of General Thoracic Surgery, Department of Surgery, Changhua Christian Hospital, Changhua, Taiwan; 6 Department of Sports Medicine, College of Health Care, China Medical University, Taichung, Taiwan; 7 Department of Orthopedic Surgery, China Medical University Beigang Hospital, Yunlin, Taiwan; 8 Department of Neurosurgery, China Medical University Hospital, Taichung, Taiwan; 9 Department of Medicine, MacKay Medical College, New Taipei City, Taiwan; 10 School of Pharmacy, Kaohsiung Medical University, Kaohsiung, Taiwan; 11 Institute of Biomedical Sciences, Mackay Medical College, Taipei, Taiwan; 12 School of Oral Hygiene, College of Oral Medicine, Taipei Medical University, Taipei, Taiwan; 13 Department of Pharmacology, School of Medicine, China Medical University, Taichung, Taiwan; 14 Chinese Medicine Research Center, China Medical University, Taichung, Taiwan; 15 Department of Medical Laboratory Science and Biotechnology, College of Health Science, Asia University, Taichung, Taiwan; 16 Department of Medical Research, China Medical University Hsinchu Hospital, Hsinchu, Taiwan

**Keywords:** ANGPTL2, VEGF-A, lung cancer, lymphangiogenesis, LEC

## Abstract

Lung cancer is an extremely common cancer and metastatic lung cancer has a greatly low survival rate. Lymphangiogenesis is essential for the development and metastasis of lung cancer. The adipokine angiopoietin-like protein 2 (ANGPTL2) regulates tumor progression and metastasis, although the functions of ANGPTL2 in lung cancer are unknown. Analysis of data from TCGA genomics program, the GEPIA web server and the Oncomine database revealed that higher levels of ANGPTL2 expression were correlated with progressive disease and lymph node metastasis. ANGPTL2 enhanced VEGF-A-dependent lymphatic endothelial cell (LEC) tube formation and migration. Integrin α5β1, p38 and nuclear factor (NF)-κB signaling mediated ANGPTL2-regulated lymphangiogenesis. Importantly, overexpression ANGPTL2 facilitated tumor growth and lymphangiogenesis *in vivo*. Thus, ANGPTL2 is a promising therapeutic object for treating lung cancer.

## INTRODUCTION

Lung cancer is one of the extremely common cancers worldwide and the resulting cause of cancer death [1]. The majority of newly diagnosed patients have locally advanced or metastatic disorder, and many who undergo surgery for early-stage disease experience recurrence within the first 5 years postoperatively [2]. Lung cancer metastasis involves several processes; the establishment of hypoxia, the formation of lymphangiogenesis and angiogenesis, cancer cell migratory and invasive activities, and the appearance of distant metastasis [3–5]. In particular, lymphangiogenesis enables lymphatic endothelial cells (LECs) to form lymphatic tubes around cancers, facilitating the invasion of cancer cells into the lymph node [6, 7]. Vascular endothelial growth factor (VEGF) reportedly enhances LEC-mediated lymphangiogenesis and lung cancer metastasis [8, 9]. Tumor-secreted VEGF family proteins, such as VEGF-A, -C and -D, are critical mediators in the regulation of LEC proliferation and promotion of lymphangiogenesis [6, 7]. Levels of VEGF production are higher in lung cancer patients than in normal healthy controls [10, 11]. Thus, it is critical to investigate the mechanisms underlying VEGF overexpression and VEGF-induced promotion of LEC-mediated lymphangiogenesis and lung cancer metastasis.

Adipokines, proteins secreted by adipocyte tissue, facilitate tumorigenesis and distant metastasis in different types of cancers [12–14]. The adipokine angiopoietin-like protein 2 (ANGPTL2) is member of the angiopoietin-like family and acts as a growth factor of vascular endothelium [15]. ANGPTL2 is greatly produced in adipose tissue and obese mice exhibit upregulated levels of ANGPTL2 mRNA and circulating protein [16]. Abnormally high levels of ANGPTL2 have been found in lung cancer cells and serum from patients with colorectal or gastric cancer [17–19], with evidence showing that upregulated ANGPTL2 expression promotes the growth, drug resistance and metastasis of colorectal cancer [18, 20]. In addition, ANGPTL2 has reported to enhance the progression and metastasis of lung cancer [21–23]. However, the regulating effects of ANGPTL2 upon lymphangiogenesis remain unknown in lung cancer. In current report, we found higher levels of ANGPTL2 and LYVE-1 (a LEC marker) expression in lung cancer tissue than in normal control tissue. We also indicate that ANGPTL2 promotes VEGF-A-dependent lymphangiogenesis via integrin α5β1, p38 MAP kinase (MAPK) and NF-κB signaling, indicating that ANGPTL2 may be worth targeting when applying tumor-associated lymphangiogenesis.

## MATERIALS AND METHODS

### Materials

ANGPTL2 (ab199133) antibody was purchased from Abcam (Cambridge, MA, USA), VEGF-A (A17877) was purchased from Abclonal (Cambridge, MA, USA), p-p65 (3033) antibody was purchased from Cell Signaling Technology (Danvers, MA, USA), β-actin (GT5512) and p65 (GTX102090) antibodies were purchased from GeneTex (Hsinchu, Taiwan). Small interfering RNAs (siRNAs) against integrin α5 (sc-29372) and β1(sc-35674), and immunoglobulin (Ig)-like transcript 4 (ILT4) (sc-45200) were purchased from Santa Cruz Biotechnology (Santa Cruz, CA, USA). VEGF-A recombinant protein was acquired from PeproTech (Rocky Hill, NJ, USA). The p38 MAPK inhibitor SB203580 (HY-10256) and NF-κB inhibitor PDTC (P8765-1G) was acquired from Sigma-Aldrich (St. Louis, MO, USA). The ANGPTL2 overexpression plasmid (ANGPTL2 cDNA) was commercially synthesized by NCFB (Academia Sinica, Taiwan). ANGPTL2 (clone ID: TRCN0000158500) knockdown (sh-ANGPTL2) was purchased from the National RNAi Core Facility (RNAi Core, Academia Sinica, Taiwan).

### Cell culture

The human lung adenocarcinoma cell line A549 was purchased from the American Type Culture Collection (ATCC; Manassas, VA, USA). CL1-0 and CL1-5 cell lines were provided by Dr. Shun-Fa Yang (Chung Shan Medical University, Taiwan). Cells were cultured in DMEM medium containing streptomycin (100 μg/mL), penicillin (100 U/mL) and 10% FBS and maintained at 37° C and 5% CO_2_.

The human LEC cell line was purchased from Lonza (Walkersville, MD, USA). Cells were grown in EGM™-2 MV BulletKit™ Medium consisting of basal medium plus the EBM™-2 MV SingleQuot™ Kit (Lonza; Walkersville, MD, USA). Cells were seeded onto 1% gelatin-coated plastic ware and cultured at 37° C and 5% CO_2_.

### Quantification of ANGPTL2 and LYVE-1 expression

Levels of ANGPTL2 and LYVE-1 expression in normal and tumor human tissue specimens were quantified using The Cancer Genome Atlas (TCGA) genomics program, the Gene Expression Profiling Interactive Analysis (GEPIA) web server and the Oncomine database [24, 25]. A total of 11 lung adenocarcinoma tissue samples from the Gene Expression Omnibus (GEO) database (GDS4402/1431848_at) were analyzed for levels of the *ANGPTL2* gene in lymph node metastatic tumor cells (n=8) and primary lung tumor cells (n=3). The online database Kaplan–Meier Plotter (http://www.kmplot.com) was used to examine the association between ANGPTL2 expression and overall survival in human lung cancer.

### Collection of lung cancer conditioned medium

Lung cancers were pretreated or transfected with the pharmacological inhibitors or genetic siRNAs, then treated with ANGPTL2. The medium was collected as conditioned medium (CM) and stored at −80° C until use.

### Measurement of LEC migration

LEC migratory activity was evaluated using Transwell inserts in 24-well dishes (Costar, NY, USA), as according to our previous research [26]. Migratory cells were imaged under ×200 magnification using an Eclipse Ti2 microscope (Nikon, Tokyo, Japan).

### Measurement of LEC tube formation

LECs (3 × 10^5^ cells) were cultured in 50% EGM™-2MV medium and 50% lung cancer CM, then applied to plates precoated with Matrigel. LEC tube formation was photographed after 6 h and the number of tube branches was counted manually [27].

### Transient transfection and luciferase assays

Lung cancer cells were transfected with integrin α5, β1 and ILT4 siRNA or nuclear factor-κB (NF-κB) luciferase plasmid (Stratagene; St. Louis, MO, USA) using Lipofectamine 2000, then treated with ANGPTL2. The Dual-luciferase^®^ Reporter Assay System was performed to analyze luciferase activity.

### Establishment of ANGPTL2 knockdown CL1-5 cells and overexpression CL1-0 cells

To establish ANGPTL2 knockdown CL1-5 cells and overexpression CL1-0 cells, a lentivirus was prepared according to a standard protocol [28]. For infection, CL1-5 and CL1-0 cells were seeded in a 6-well dish and the lentivirus was added to the medium (multiplicity of infection = 10). After 24 h, the culture medium was changed and then at 48 h, 2 μg/mL of puromycin was added to select for ANGPTL2 knockdown and overexpression cells.

### Immunohistochemistry (IHC) staining

The tissues were stained with VEGF-A or LYVE-1 antibodies and quantified according to the protocol described in our previous work [29, 30]. The sum of the intensity and percentage scores was used as the final staining score, as described previously [25].

### Statistical analysis

All values are expressed as the mean ± standard deviation (S.D.). Statistical differences between the experimental groups were assessed for significance using the Student’s *t*-test. Statistical comparisons of more than two groups were performed using one-way analysis of variance (ANOVA) with the Bonferroni post hoc test. Between-group differences were considered to be significant if the *p*-value was less than 0.05.

Materials and Methods relating to western blot assay, reverse transcription-quantitative PCR (RT-qPCR) assay and tumor xenograft study are all obtainable within Supplementary Information.

### Data availability statement

The data sets used and analyzed during the current study are available from the corresponding author on reasonable request.

## RESULTS

### ANGPTL2 is highly expressed in patients with progressive lung cancer disease and lymph node metastasis

**Figure 1:**
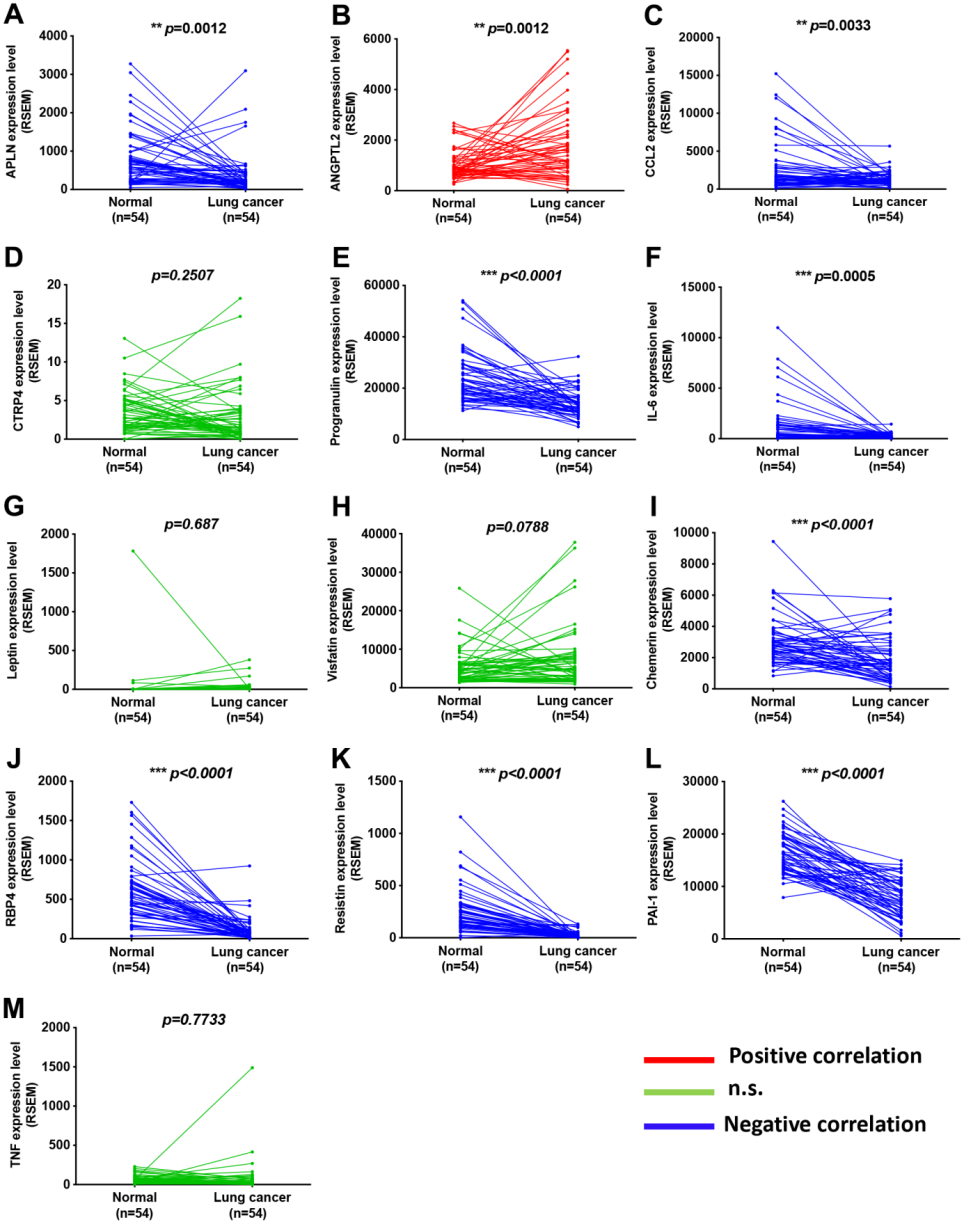
**Adipokine levels in lung cancer tissue and normal healthy samples.** (**A**–**M**) Adipokine mRNA expression in human lung cancer tissue and adjacent normal tissue was analyzed in records from the TCGA database. *p < 0.05 compared with normal tissue.

**Figure 2:**
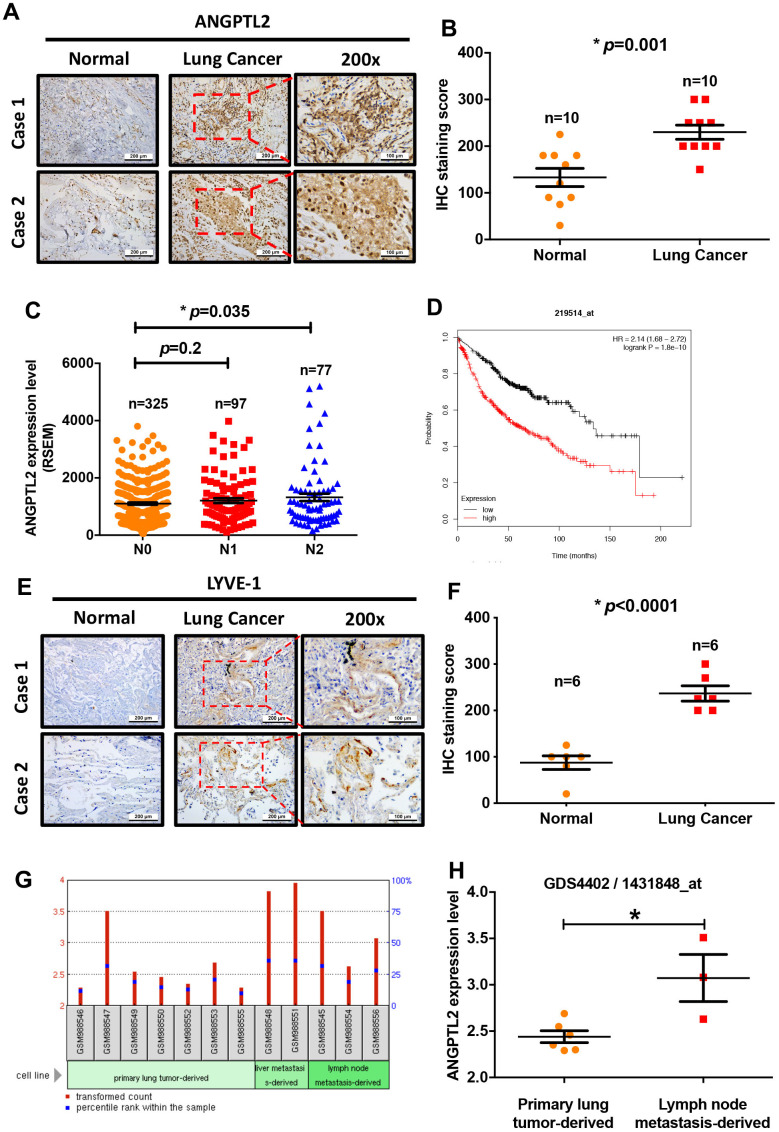
**Levels of ANGPTL2 expression correlate with clinicopathologic features of lung adenocarcinoma tissue infiltrated by lymphatic vessels.** (**A**, **B**) ANGPTL2 expression in human lung cancer tissue and adjacent normal tissue samples was analyzed by IHC staining. (**C**) The association between ANGPTL2 expression and regional lymph node metastasis was analyzed in samples from the TCGA database. (**D**) Associations between ANGPTL2 expression and overall survival rates of lung cancer patients were analyzed using the Kaplan-Meier Plotter database. (**E**, **F**) LYVE-1 expression in human lung cancer tissue and adjacent normal tissue samples was analyzed by IHC staining. (**G**, **H**) Data obtained from the GEO database (GDS4402/1431848_at) were analyzed for ANGPTL2 expression in primary lung tumor tissue and lung cancer with lymph node metastasis tissue samples. *p < 0.05 compared with normal tissue.

Adipokines can promote cancer progression and metastasis [12–14]. To examine the effects of adipokine levels upon lung cancer progression, we investigated the clinical significance 13 of adipokines identified in lung adenocarcinoma samples from the TCGA database. We found higher levels of ANGPTL2 mRNA expression in tumor tissue than in adjacent normal tissue (Figure 1). Conversely, levels of apelin, CCL2, progranulin, interleukin-6, chemerin, retinol binding protein 4 (RBP4), resistin, and plasminogen activator inhibitor-1 (PAI-1) expression were lower in tumor tissue than in adjacent normal tissue (Figure 1). Thus, ANGPTL2 is a more important adipokine than others in lung cancer progression. Similarly, IHC data confirmed upregulated expression of ANGPTL2 in lung cancer tissue (Figure 2A, 2B), while TCGA data revealed significant associations between high levels of ANGPTL2 expression and regional lymph node metastasis (Figure 2C). Furthermore, higher levels of ANGPTL2 expression in patients with lung adenocarcinoma were associated with lower survival (Figure 2D).

Cancer cells increase lymphatic vessel density in the tumor microenvironment and in lymph nodes by secreting lymphangiogenic factors, thereby promoting lymph node metastasis and poor prognosis [31, 32]. To characterize lymphatic vessels in lung tumors, we used an IHC assay with LYVE-1 antibody. This detected intratumor lymphatic vessels in the tumor peripheral region of lung cancer patients and none in tissue from normal healthy controls (Figure 2E, 2F). Records from the Gene Expression Omnibus (GEO) database (GDS4402/1431848_at) showed higher ANGPTL2 levels in lymph node metastatic tumor cells than in primary lung tumor cells from animal lung adenocarcinoma tissue (Figure 2G, 2H). Our results indicate that high levels of ANGPTL2 are positively associated with poor survival and lymph node metastasis in lung cancer.

### ANGPTL2 facilitates VEGF-A-dependent LEC tube formation and migration

**Figure 3:**
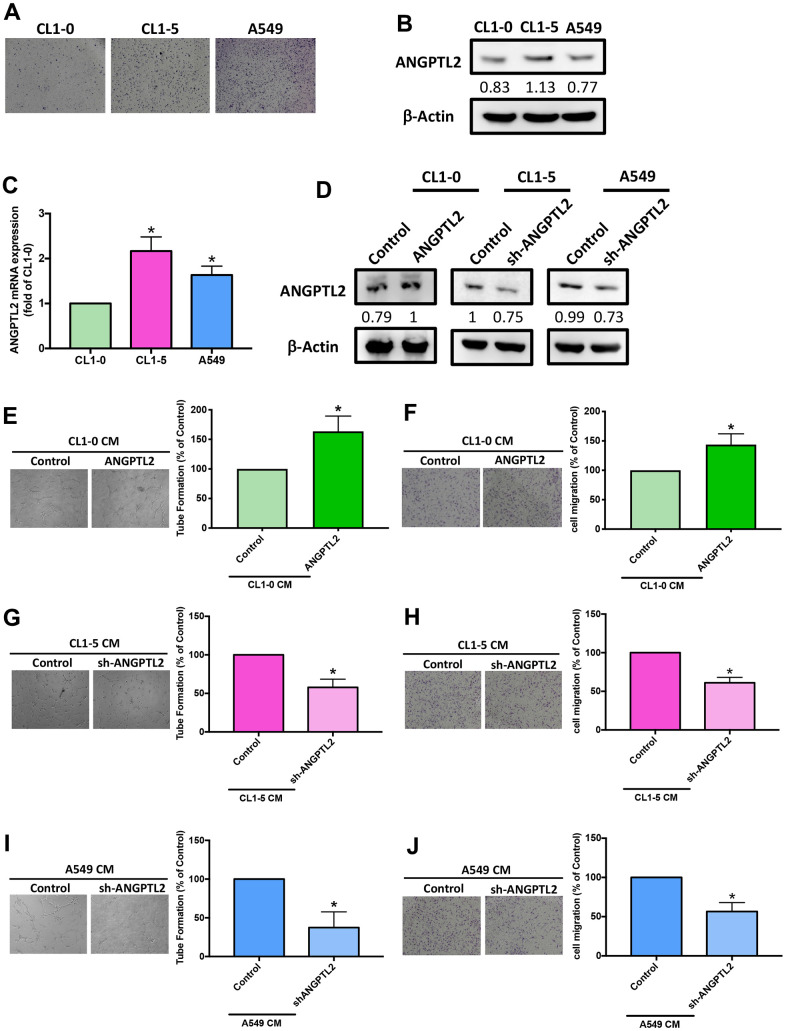
**ANGPTL2 levels correlate with lung cancer cell migratory activity and facilitate LEC tube formation and migration.** (**A**) The migratory ability of human lung cancer cell lines (CL1-0, CL1-5 and A549) was measured by the Transwell assay. (**B**, **C**) ANGPTL2 mRNA and protein expression in lung cancer cell lines was examined by Western blot (n=3) and qPCR. (**D**) Cells were transfected with ANGPTL2 cDNA or shRNA, then ANGPTL2 expression was measured by Western blot (n=3). *p < 0.05 compared with CL1-0 cells. (**E**–**J**) Cells were transfected with ANGPTL2 cDNA or shRNA. The CM was collected and applied to the LECs. LEC tube formation and migration was examined. *p < 0.05 compared with controls.

To examine the effects of ANGPTL2 in lung cancer lymph node metastasis, we measured basal migratory activities of lung cancer cell lines CL1-0, CL1-5, and A549. CL1-5 displayed higher migratory ability than A549 and CL1-0 (Figure 3A). Levels of ANGPTL2 protein and mRNA expression were also higher in CL1-5 cells than in A549 and CL1-0 cells (Figure 3B, 3C), implying that ANGPTL2 level is associated with migratory ability in lung cancer cells. After transfecting CL1-0 cells with ANGPTL2 cDNA, ANGPTL2 expression increased (Figure 3D). *In vitro* LEC tube formation and migration is a well-established model for mimicking lymphangiogenesis [7]. CM from ANGPTL2 cDNA-transfected lung cancer cells markedly facilitated tube formation and migration in LECs (Figure 3E, 3F), while transfecting CL1-5 and A549 cells with ANGPTL2 shRNA reduced ANGPTL2 expression, LEC tube formation and migration (Figure 3D, 3G–3J), indicating that ANGPTL2 promotes lymphangiogenesis in lung cancer cells.

**Figure 4:**
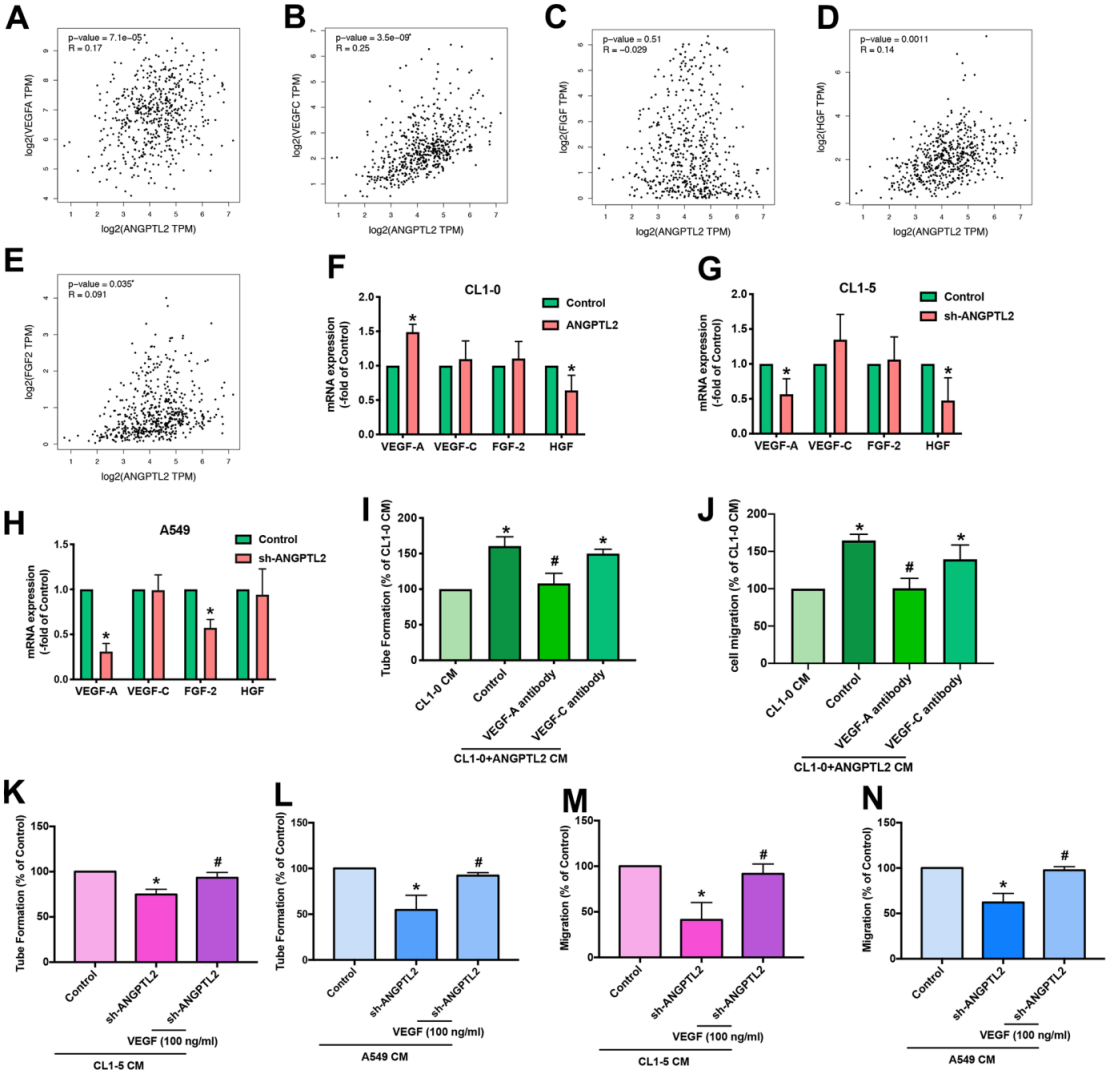
**ANGPTL2 promotes VEGF-A-dependent LEC tube formation and migration.** (**A**–**E**) Associations between ANGPTL2 expression and lymphangiogenic factors (VEGF-A, VEGF-C, FIGF, HGF and FGF2) were analyzed using data from the TCGA database. (**F**–**H**) CL1-0 cells were transfected with ANGPTL2 cDNA; CL1-5 and A549 cells were transfected with ANGPTL2 shRNA. Levels of mRNA expression were examined by qPCR. *p < 0.05 compared with controls. (**I**, **J**) CL1-0 cells were transfected with ANGPTL2 cDNA. The CM was collected and applied to the LECs with VEGF-A or VEGF-C antibody. LEC tube formation and migration was examined. (**K**–**N**) Cells were transfected with ANGPTL2 shRNA. The CM was collected and applied to the LECs with VEGF-A. LEC tube formation and migration was examined. *p < 0.05 compared with CL1-0 CM; #*p* < 0.05 compared with Control; #*p* < 0.05 compared with CL1-5 or A549 with ANGPTL2 shRNA CM.

We next investigated lymphangiogenic factors in ANGPTL2-mediated effects. Records from the TCGA database revealed that VEGF-A, VEGF-C, hepatocyte growth factor (HGF) and fibroblast growth factor-2 (FGF-2), but not C-fos-induced growth factor (FIGF), were positively correlated with ANGPTL2 expression (Figure 4A–4E). Overexpression and knockdown of ANGPTL2 markedly regulated VEGF-A synthesis but not that of other growth factors (Figure 4F–4H). Antibody to VEGF-A, but not to VEGF-C, antagonized ANGPTL-2-induced LEC lymphangiogenesis (Figure 4I, 4J and Supplementary Figure 1). Notably, treatment with VEGF-A reversed ANGPTL-2 shRNA-induced reductions in LEC tube formation and migration (Figure 4K–4N), implying that ANGPTL2 facilitates VEGF-A-dependent lymphangiogenesis in lung cancer cells.

### Integrin α5β1, p38 and NF-κB signaling pathway is mediated ANGPTL2-facilitated VEGF-A expression

**Figure 5:**
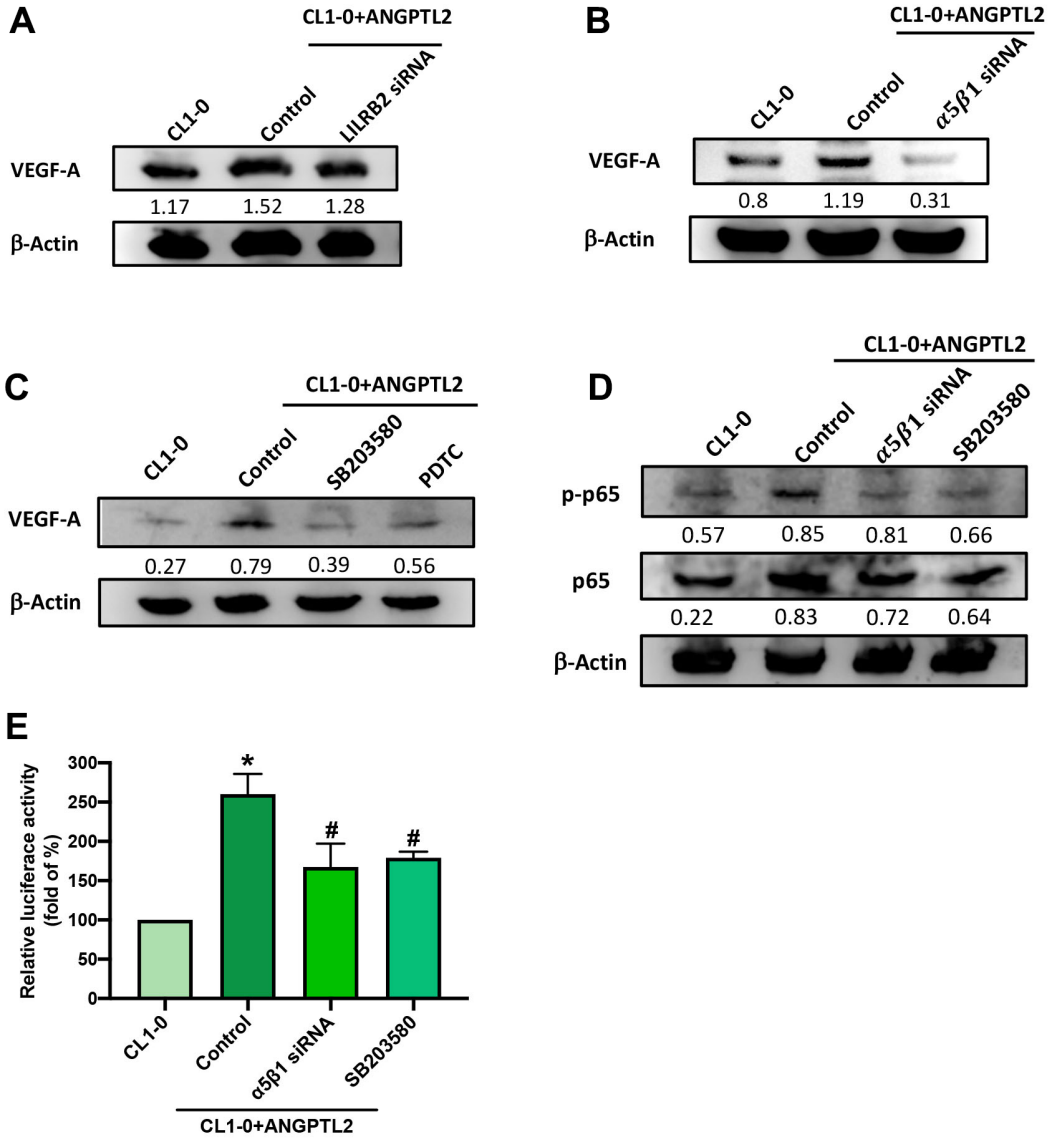
**ANGPTL2 increases VEGF-A synthesis in lung cancer cells via the integrin α5β1 receptor, p38 and NF-κB signaling.** (**A**–**C**) Cells were transfected with ANGPTL2 cDNA, then stimulated with LILRB2 and integrin α5β1 siRNA or SB203580 and PDTC; VEGF-A expression was measured by Western blot (n=3). Quantitative data of the protein level were obtained using ImageJ software. Densitometric analysis of protein expression was normalized to β-actin. (**D**, **E**) Cells were transfected with ANGPTL2 cDNA, then stimulated with integrin α5β1 siRNA and SB203580; p38 and NF-κB activation was measured by Western blot (n=3) and NF-κB luciferase activity. Quantitative data of the protein level were obtained using ImageJ software. Densitometric analysis of protein expression was normalized to β-actin. *p < 0.05 compared with CL1-0; #*p* < 0.05 compared with Control.

Leukocyte immunoglobulin-like receptor B2 (LILRB2) and integrin α5β1 are major functional receptors of ANGPTL2 [15]. Transfection of CL1-0 cells with integrin α5β1, but not LILRB2 siRNA, antagonized ANGPTL2-induced increases in VEGF-A expression (Figure 5A, 5B). The p38/NF-κB pathway regulates ANGPTL2-mediated cellular functions [16]. Treatment with p38 (SB203580) and NF-κB (PDTC) inhibitors both prevented ANGPTL2-induced increases in VEGF-A synthesis (Figure 5C). Integrin α5β1 siRNA and SB203580 reduced ANGPTL2-induced promotion of p65 phosphorylation and NF-κB luciferase activity (Figure 5D, 5E). Thus, ANGPTL2 enhances VEGF-A synthesis in lung cancer cells via integrin α5β1, p38 and NF-κB signaling.

### ANGPTL2 promotes lymphangiogenesis *in vivo*

**Figure 6:**
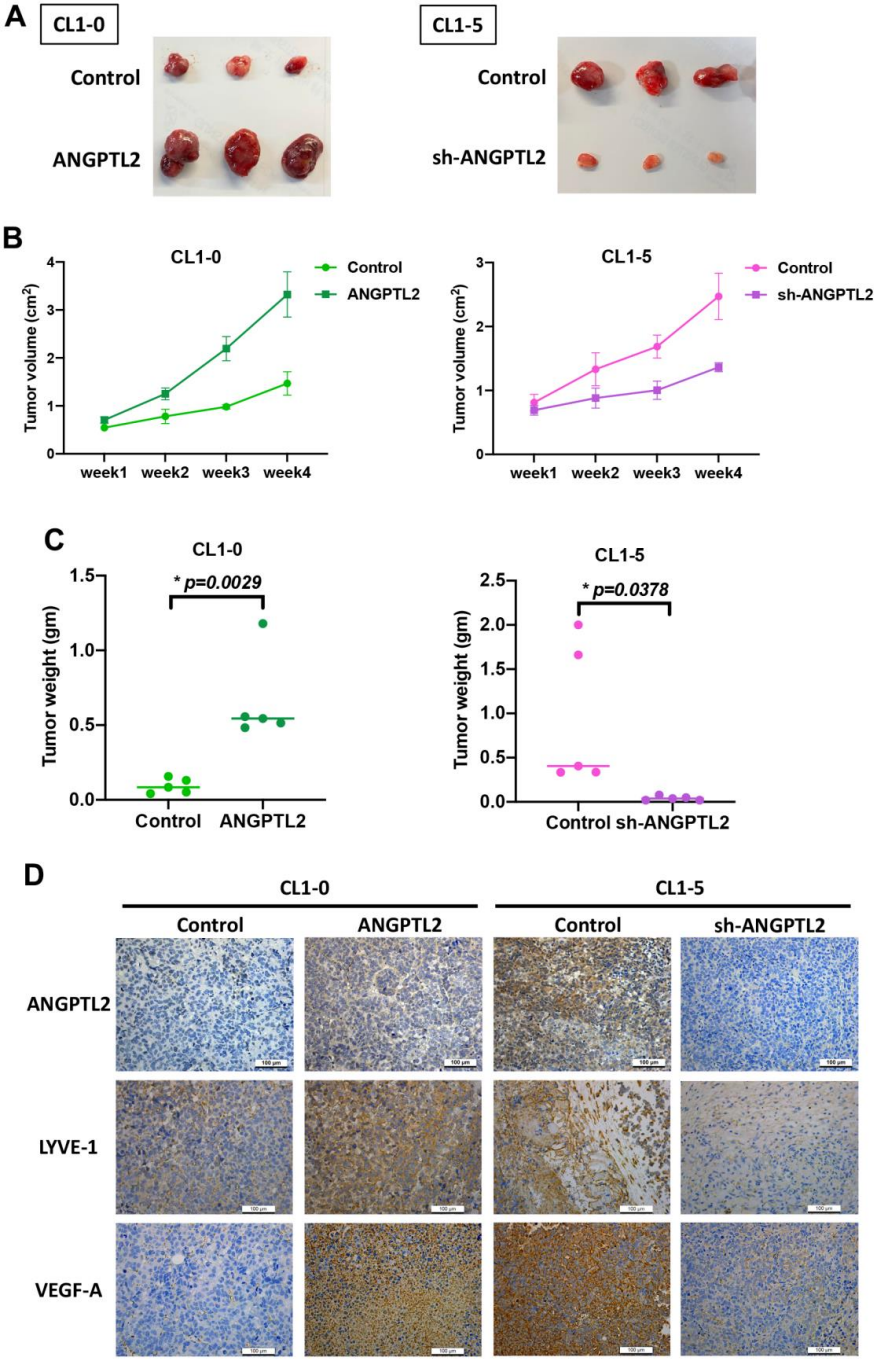
**ANGPTL2 promotes tumor growth and lymphangiogenesis *in vivo*.** (**A**–**C**) CL1-0 and CL1-5 cells were subcutaneously injected into the right flanks of BALB/c-nu mice. Four weeks later, the mice were sacrificed and the tumors were excised and weighed (n=5). (**D**) Tumor sections were immunostained using ANGPTL2, LYVE-1 and VEGF-A antibodies. *p < 0.05 compared with controls.

Next, we examined whether ANGPTL2 regulates lung cancer lymphangiogenesis in tumor xenograft mouse models. Overexpression of ANGPTL2 in CL1-0 cells increased tumor growth (Figure 6A–6C). IHC staining demonstrated that ANGPTL2 overexpression significantly increased the expression of ANGPTL2, LYVE-1 and VEGF-A (Figure 6D). In contrast, ANGPTL2 blockade suppressed tumor growth and the levels of ANGPTL2, LYVE-1 and VEGF-A (Figure 6). These results support the targeting of ANGPTL-2 for regulating tumor growth and lymphangiogenesis in lung cancer.

## DISCUSSION

It is well established that advanced lung cancer is extremely aggressive and associated with metastasis [33]. Lymphangiogenesis is essential for the development and metastasis of lung cancer [34]. Understanding the underlying mechanisms of lung cancer lymphangiogenesis may assist with the development of novel treatment approaches for lung cancer, which has a low overall five-year survival rate, despite improvements in surgical management and therapeutic combinations of radiation and chemotherapy. The adipokine ANGPTL2 is associated with cancer progression and metastasis [21, 22]. High levels of ANGPTL2 have been found in lung and gastric cancer patients [17–19]. In addition, ANGPTL2 enhanced the progression and drug resistance in colorectal cancer [18, 20]. We report finding high levels of ANGPTL2 expression in human specimens of lymph node metastatic lung cancer. Upregulation of ANGPTL2 indicates a poor prognosis with reduced survival in lung cancer patients. Our findings show that ANGPTL2 facilitates VEGF-A-dependent LEC lymphangiogenesis in lung cancer cells and that the integrin α5β1, p38 and NF-κB signaling cascade is involved in ANGPTL2-enhanced promotion of VEGF-A synthesis. Importantly, ANGPTL2 promoted lung cancer growth and lymphangiogenesis *in vivo*. Thus, ANGPTL2 is a promising therapeutic target for treating lung cancer progression and metastasis.

VEGF-A, VEGF-C and VEGF-D are major lymphangiogenic factors that regulate lymphangiogenic progression during tumor development and metastasis [6, 7, 35]. Amongst these growth factors, VEGF-C is strongly associated with the control of lymphatic vessel invasion and lymphangiogenesis [36]. In this study, overexpression or knockdown of ANGPTL2 significantly regulated VEGF-A production but not that of VEGF-C, HGF, or FGF-2. ANGPTL2-induced facilitation of LEC tube formation and migration was antagonized by treatment with VEGF-A antibody, but not VEGF-C antibody. Recombinant VEGF-A rescued the inhibition of LEC lymphangiogenesis following ANGPTL2 blockade. Our mouse xenograft model revealed that ANGPTL2 overexpression promotes the upregulation of LYVE-1 and VEGF-A expression in lung cancer tissue. Thus, ANGPTL2 promotes VEGF-A-dependent LEC lymphangiogenesis. Similarly, VEGF-A reportedly mediates simvastatin-induced regulation of tumor lymphangiogenesis and lymph node metastasis [37]; recombinant canstatin suppresses VEGF-A-mediated lymphangiogenesis in an animal model of oral squamous cell carcinoma [38]. Thus, VEGF-A is a critical regulator of lymphangiogenesis during tumor growth and metastasis.

A previous report has indicated that ANGPTL2-induced regulation of cellular functions occurs via LILRB2 and the integrin α5β1 receptor [15]. When this study used siRNAs against LILRB2 and integrin α5β1, we found that integrin α5β1 siRNA but not LILRB2 siRNA antagonized ANGPTL2-facilitated VEGF-A production, suggesting that the integrin α5β1 receptor controls ANGPTL2-enhanced promotion of VEGF-A synthesis and lymphangiogenesis. It is known that p38 activation is crucial for regulating different cellular events [39], such as the promotion of lymphangiogenesis and metastasis [39, 40, 41]. Our data found that ANGPTL2 enhances p38 phosphorylation, while the p38 inhibitor reversed ANGPTL2-regulated VEGF-A production. NF-κB signaling is an important downstream molecule (or mediator) of p38 in the regulation of ANGPTL2-induced inflammatory responses [16]. Our data showed that the NF-κB inhibitor antagonized ANGPTL2-mediated VEGF-A synthesis in lung cancer cells. Our results also showed that ANGPTL2 enhances p65 phosphorylation and NF-κB luciferase activity, which was reduced by the integrin α5β1 siRNA and p38 inhibitor, indicating that integrin α5β1 receptor-dependent p38/NF-κB activation regulates ANGPTL2-induced mediation of VEGF-A expression and lymphangiogenesis in human lung cancer cells.

**Figure 7:**
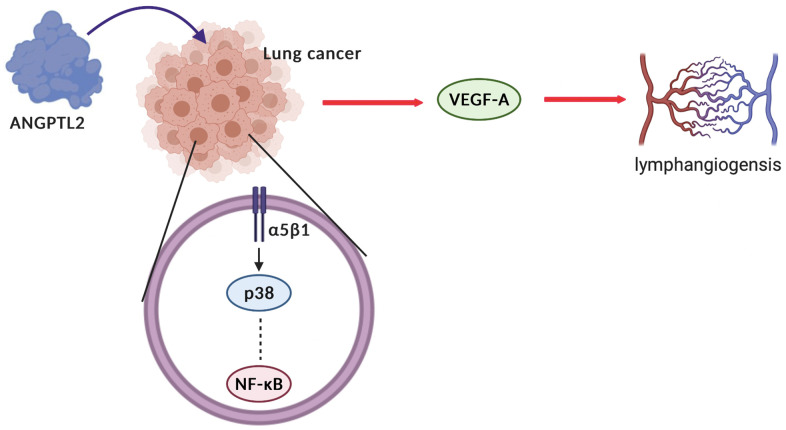
**Schematic diagram summarizes the mechanisms by which ANGPTL2 facilitates lymphangiogenesis in human lung cancer cells.** ANGPTL2 increases VEGF-A production and subsequently facilitates LEC lymphangiogenesis in human lung cancer cells via the integrin α5β1 receptor, p38 and NF-κB signaling.

The limitations should be noted in this study. The study results could have been strengthened statistically by using more lung cancer patient tissues. In addition, the more detail clinicopathologic data (age, sex and pathology diagnoses) should be enrolled. Secondly, although our data strongly suggest that ANGPTL2 promotes VEGF-A-mediated lymphangiogenesis in human lung cancer cells, we cannot exclude the possibility that ANGPTL2 also promotes the activities of other angiogenetic factors. In summary, our study indicates that ANGPTL2 promotes VEGF-A synthesis in human lung cancer cells and subsequently facilitates LEC lymphangiogenesis via the integrin α5β1 receptor, p38 and NF-κB signaling (Figure 7). These results improve our understanding about how ANGPTL2-mediated VEGF-A synthesis contributes to lymphangiogenesis, which may help scientists design more effective therapy for metastatic lung cancer.

## Supplementary Material

Supplementary Materials and Methods

Supplementary Figure 1
