# Supplementary material for: ANGPTL2 promotes VEGF-A synthesis in human lung cancer and facilitates lymphangiogenesis
Source: Aging (Albany NY). 2023 Mar 13;15(5):1652–67. doi: 10.18632/aging.204581 (PMC10042695; doi:10.18632/aging.204581)
Supplement: Supplementary Materials and Methods [file aging-15-204581-s001.pdf]

SUPPLEMENTARY MATERIALS

Supplementary Materials and Methods

Real-time quantitative PCR analysis

Total RNA was isolated from lung cancer cells using TRIzol reagent (MDBio; Taipei, Taiwan). RNA (1 μg) was reverse-transcribed into cDNA with oligo-DT primer, according to the manufacturer's procedure (Invitrogen; Carlsbad, CA, USA). qPCR was performed using SYBR Green with sequence-specific primers (Invitrogen; Carlsbad, CA, USA). qPCR assays were performed with StepOnePlus (Applied Biosystems; Foster City, CA, USA) [1, 2].

Western blot

Lung cancer cells were treated with RIPA buffer. Isolated proteins were subjected to SDS-PAGE and transferred to polyvinylidene difluoride membranes (Merck; Darmstadt, Germany) [3, 4]. The membranes were blocked with 5% non-fat milk, treated with primary antibodies, then washed and treated with secondary antibodies, before being visualized using the ImageQuant™ LAS 4000 biomolecular imager [5–7].

Tumor xenograft study

Lung cancer cells (5×10^6) were transplanted subcutaneously into the right flanks of BALB/c-nu mice (4-week-old males), following a previously detailed method [8]. After 4 weeks, the mice were sacrificed by CO_2 inhalation and the tumors were removed. All animal work was carried out in accordance with a protocol approved by the Institutional Animal Care and Use Committee of China Medical University (Taichung, Taiwan).

Supplementary References

1. Lee HP, Wu YC, Chen BC, Liu SC, Li TM, Huang WC, Hsu CJ, Tang CH. Soya-cerebroside reduces interleukin production in human rheumatoid arthritis synovial fibroblasts by inhibiting the ERK, NF-κB and AP-1 signalling pathways. Food and Agricultural Immunology. 2020; 31:740–50. https://doi.org/10.1080/09540105.2020.1766426

2. Lee HP, Liu SC, Wang YH, Chen BC, Chen HT, Li TM, Huang WC, Hsu CJ, Wu YC, Tang CH. Cordycerebroside A suppresses VCAM-dependent monocyte adhesion in osteoarthritis synovial fibroblasts by inhibiting MEK/ERK/AP-1 signaling. Journal of Functional Foods. 2021; 86:104712. https://doi.org/10.1016/j.jff.2021.104712

3. Lee HP, Wang SW, Wu YC, Lin LW, Tsai FJ, Yang JS, Li TM, Tang CH. Soya-cerebroside inhibits VEGF-facilitated angiogenesis in endothelial progenitor cells. Food and Agricultural Immunology. 2020; 31:193–204. https://doi.org/10.1080/09540105.2020.1713055

4. Cheng FJ, Huynh TK, Yang CS, Hu DW, Shen YC, Tu CY, Wu YC, Tang CH, Huang WC, Chen Y, Ho CY. Hesperidin Is a Potential Inhibitor against SARS-CoV-2 Infection. Nutrients. 2021; 13:2800. https://doi.org/10.3390/nu13082800 PMID:34444960

5. Lee HP, Wang SW, Wu YC, Tsai CH, Tsai FJ, Chung JG, Huang CY, Yang JS, Hsu YM, Yin MC, Li TM, Tang CH. Glucocerebroside reduces endothelial progenitor cell-induced angiogenesis. Food and Agricultural Immunology. 2019; 301033–45. https://doi.org/10.1080/09540105.2019.1660623

6. Su CM, Tang CH, Chi MJ, Lin CY, Fong YC, Liu YC, Chen WC, Wang SW. Resistin facilitates VEGF-C-associated lymphangiogenesis by inhibiting miR-186 in human chondrosarcoma cells. Biochem Pharmacol. 2018; 154:234–42. https://doi.org/10.1016/j.bcp.2018.05.001 PMID:29730230

7. Wu KM, Hsu YM, Ying MC, Tsai FJ, Tsai CH, Chung JG, Yang JS, Tang CH, Cheng LY, Su PH, Viswanadha VP, Kuo WW, Huang CY. High-density lipoprotein ameliorates palmitic acid-induced lipotoxicity and oxidative dysfunction in H9c2 cardiomyoblast cells via ROS suppression. Nutr Metab (Lond). 2019; 16:36. https://doi.org/10.1186/s12986-019-0356-5 PMID:31149020

8. Lin CY, Tzeng HE, Li TM, Chen HT, Lee Y, Yang YC, Wang SW, Yang WH, Tang CH. WISP-3 inhibition of miR-452 promotes VEGF-A expression in chondrosarcoma cells and induces endothelial progenitor cells angiogenesis. Oncotarget. 2017; 8:39571–81. https://doi.org/10.18632/oncotarget.17142 PMID:28465477
