# Supplementary material for: ANGPTL2 promotes VEGF-A synthesis in human lung cancer and facilitates lymphangiogenesis
Source: Aging (Albany NY). 2023 Mar 13;15(5):1652–67. doi: 10.18632/aging.204581 (PMC10042695; doi:10.18632/aging.204581)
Supplement: Supplementary Figure 1 [file aging-15-204581-s002.pdf]

SUPPLEMENTARY FIGURE

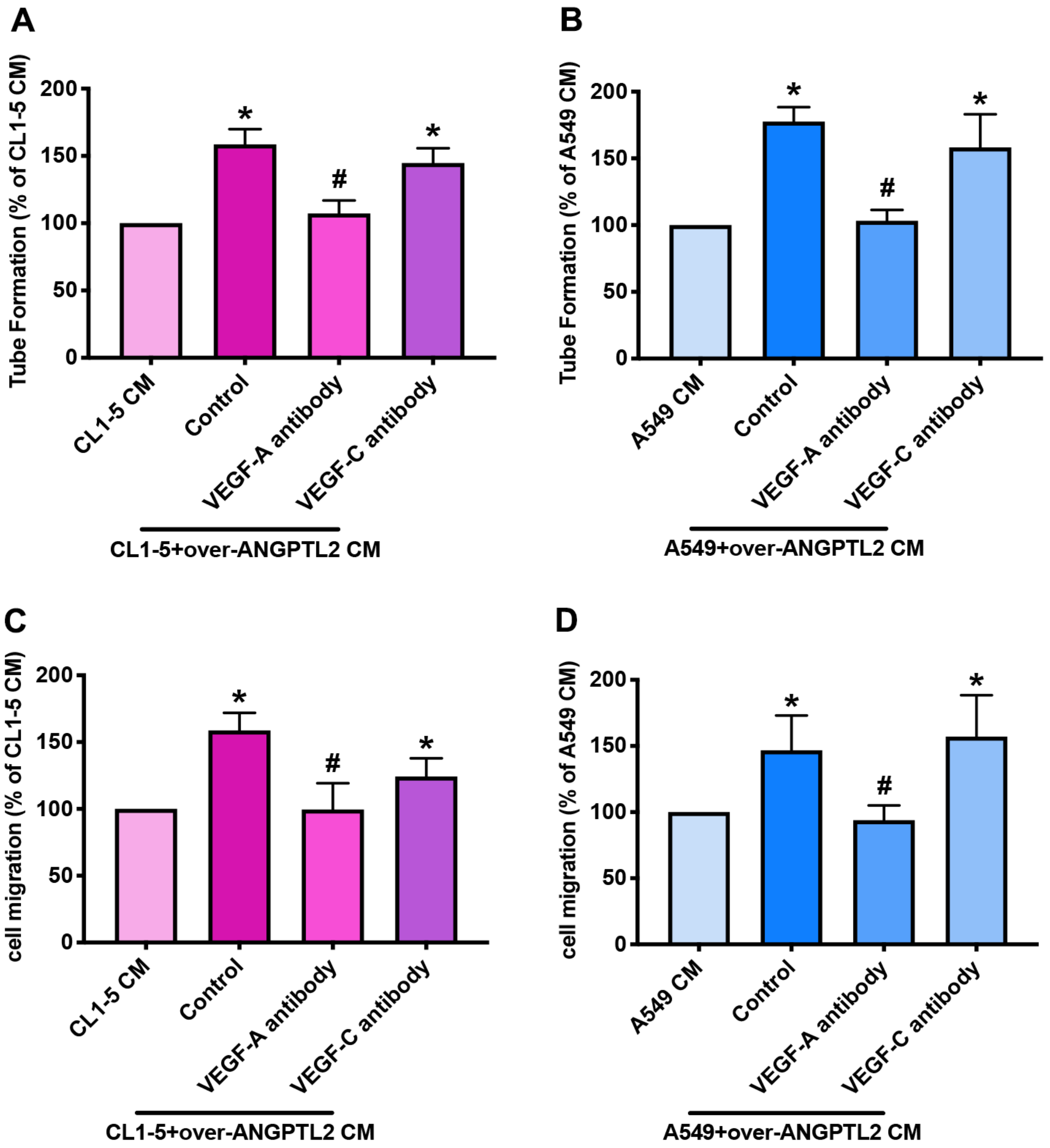

Supplementary Figure 1. ANGPTL2 promotes VEGF-A-dependent LEC lymphangiogenesis. (**A**–**D**) CL1-5 and A549 cells were transfected with ANGPTL2 shRNA. The CM was collected and applied to the LECs with VEGF-A or VEGF-C antibody. LEC tube formation and migration was examined. *$p < 0.05$ compared with CL1-5 CM or A549 CM; #$p < 0.05$ compared with Control.
